# Cocoa Supplementation Alleviates Gliadin-Induced Intestinal Dysbiosis in a Mouse Model of Celiac Disease

**DOI:** 10.3390/foods15020370

**Published:** 2026-01-20

**Authors:** Marina Girbal-González, María José Rodríguez-Lagunas, Arturo Rodríguez-Banqueri, Ulrich Eckhard, Francesc Xavier Gomis-Rüth, Àngels Franch-Masferrer, Francisco José Pérez-Cano

**Affiliations:** 1 Section of Physiology, Department of Biochemistry and Physiology, Faculty of Pharmacy and Food Science, University of Barcelona, Av. Joan XXIII, 27-31, 08028 Barcelona, Catalonia, Spain; marinagirbal@ub.edu (M.G.-G.); mjrodriguez@ub.edu (M.J.R.-L.); angelsfranch@ub.edu (À.F.-M.); 2 Research Institute of Nutrition and Food Safety (INSA-UB), University of Barcelona, Av. Prat de la Riba, 171, 08921 Santa Coloma de Gramenet, Catalonia, Spain; 3 Proteolysis Laboratory, Department of Structural and Molecular Biology, Molecular Biology Institute of Barcelona (IBMB), Higher Scientific Research Council (CSIC), Barcelona Science Park, c/Baldiri Reixac, 15-21, 08028 Barcelona, Catalonia, Spain; arbcri@ibmb.csic.es (A.R.-B.); xgrcri@ibmb.csic.es (F.X.G.-R.); 4 Synthetic Structural Biology, Department of Structural and Molecular Biology, Molecular Biology Institute of Barcelona (IBMB), Higher Scientific Research Council (CSIC), Barcelona Science Park, c/Baldiri Reixac, 15-21, 08028 Barcelona, Catalonia, Spain; ueccri@ibmb.csic.es

**Keywords:** celiac disease (CeD), polyphenols, cocoa, bioactives, PICRUSt2, microbiota, functional prediction, goblet cells

## Abstract

Celiac disease (CeD) is a chronic immune-mediated enteropathy triggered by dietary gluten in genetically predisposed individuals which also entails intestinal dysbiosis. This hallmark microbial imbalance provides a rationale for exploring interventions that could modulate the gut ecosystem. Cocoa is a bioactive food rich in polyphenols, theobromine, and fiber, compounds known to have an influence on both immune function and gut microbiota composition. Here, we investigated the effects of cocoa supplementation on the gut microbial profile and predicted functionality in DQ8-D^d^-villin-IL-15tg mice, genetically predisposed to CeD. Animals were assigned to a reference group receiving a gluten-free diet (GFD), a gluten-containing diet group (GLI), or the latter supplemented with defatted cocoa (GLI + COCOA) for 25 days. The cecal microbiota was analyzed via 16S rRNA sequencing, and functional pathways were inferred using PICRUSt2. Goblet cell counts and CeD-relevant autoantibodies were measured and correlated with microbial taxa. Cocoa supplementation partially attenuated gluten-induced dysbiosis, preserving beneficial taxa such as *Akkermansia muciniphila* and *Lactobacillus* species while reducing opportunistic and pro-inflammatory bacteria. Functional predictions suggested differences in the predicted microbial metabolic potential related to amino acid, vitamin, and phenolic compound metabolism. Cocoa also mitigated goblet cell loss and was inversely associated with anti-gliadin IgA levels. These findings suggest that cocoa, as an adjuvant to a GFD, could be of help in maintaining microbial homeostasis and intestinal health in CeD, supporting further studies to assess its translational potential.

## 1. Introduction

Celiac disease (CeD) is a chronic small intestinal immune-mediated enteropathy precipitated by exposure to dietary gluten in genetically predisposed individuals [1]. Its global prevalence is estimated to be around 1.4%, and it affects 1.5 times more women than men and twice more children than adults [2]. Nevertheless, there is currently no treatment available for CeD other than the strict adherence to a gluten-free diet (GFD), which can be burdensome from both social and economic perspectives [3]. Moreover, additional gluten-related disorders, such as non-celiac gluten/wheat sensitivity or wheat/gluten allergy, also exhibit high global prevalences, estimated to affect 1–13% [4,5] and 0.5–1% [6] of the worldwide population, respectively. To this end, there is an urgent need to uncover therapeutic alternatives or adjuvants to a GFD.

Cocoa beans (*Theobroma cacao* L. seeds) contain multiple bioactive compounds, including polyphenols (especially flavanols, anthocyanidins and proanthocyanidins), methylxanthines (theobromine and caffeine), phytosterols, and fiber [7]. As a result, cocoa has been linked to many health benefits, including modulation of the immune response, anti-carcinogenic properties, or prevention of metabolic alterations [8,9].

Recently, multiple studies have investigated the role of polyphenols in the context of CeD, and it has been proposed that polyphenols could exert a preventive effect on CeD pathogenesis due to their antioxidant and anti-inflammatory effects. However, the protective activity could stem from other mechanisms: the sequestration of gluten immunogenic peptides (GIPs) by polyphenols—thereby reducing their bioavailability—and the modulation of the gut microbial ecosystem [10]. While previous works have focused on polyphenol-containing compounds such as green tea [11], grapes [12] or propolis [13], very little is known about the potential effects of cocoa on the pathophysiology of CeD, as well as on its influence on the composition and functions of the gut microbial ecosystem in this disease.

The gut microbiota, which comprises the living microorganisms inhabiting the large intestine, has been recognized as a key regulator of both human health and disease. Increasing evidence indicates that, through its metabolites, it is extensively interconnected with multiple organs, among which the gut–brain axis is one of the most important pathways [14]. Due to its capacity to shape and modulate the immune system, the gut microbiota has been tightly related to autoimmune diseases, and especially those centered on the gastrointestinal system, as is CeD. Although the specific mechanisms are yet to be elucidated, it has been proposed that the gut microbiota can contribute to the onset of CeD through the promotion of a loss of tolerance to gluten or the activation of the immune system [15]. Moreover, another key question is whether the dysbiosis observed in CeD patients is the cause or the result of the disease [16].

Since many of the bioactive compounds present in cocoa demonstrate poor bioavailability, most of them are not absorbed in the upper gastrointestinal tract and therefore they reach the large intestine (90–95%). There, they are utilized by the resident microbes to produce primary or secondary metabolites, including short-chain fatty acids (SCFAs), indoles, or phenyl derivatives, which are then absorbed into the system and exert beneficial functions. Moreover, due to the prebiotic-like effect of cocoa fiber and procyanidins, cocoa intake has been related to an increase in the abundance of beneficial gut bacteria such as *Lactobacillus* or *Bifidobacterium* and a decrease in pathogenic/opportunistic taxa such as *Clostridium perfringens* [17]. Interestingly, previous studies on the gut microbial modulation by cocoa have reported a promotion of oral tolerance in an allergy model [18], an alteration of the microbial profile without disease remission in a model of colitis [19], or an improvement in glucose metabolism in diabetic animals [20].

To this end, in this study, we have aimed to elucidate the benefits exerted by cocoa intake in a mouse model of CeD-induced microbial dysbiosis; but, even if a beneficial modulation of the gut microbiota was achieved, any potential application in humans would be limited to an adjunct to the GFD, which remains the only accepted treatment for CeD. Hence, the objective of cocoa intake would be to counteract the microbial dysbiosis associated with CeD, thereby contributing to the alleviation of gastrointestinal symptoms and the regulation of altered biological and metabolic functions.

## 2. Materials and Methods

### 2.1. Experimental Design and Mouse Model

The animals used in this study were DQ8-D^d^-villin-IL-15tg mice, which have been described to act as a CeD model [21]. The progenitors were generously provided by V. Abadie (The University of Chicago) [22] and a colony was maintained until enough animals were obtained. In order to verify both the DQ8 transgenic phenotype and the presence of the D^d^-IL-15 and villin-IL-15 transgenes in both received and newborn mice, staining of mesenteric lymph node (MLN) lymphocytes and ear tissue multiplex PCR were performed as previously described [21]. Mice were housed in polycarbonate cages within the Animal Facility of the Universitat de Barcelona (Unitat d’Experimentació Animal de Diagonal-9900030, Barcelona, Spain), with ad libitum access to water and a gluten-free diet (GFD; CA. 170481 AIN-76A Purified Diet, Envigo, Madison, WI, USA), under controlled temperature and humidity, and a 12:12 h light–dark cycle (Ethics Committee on Animal Experimentation (CEEA) Ref. 186/20).

**Figure 1:**
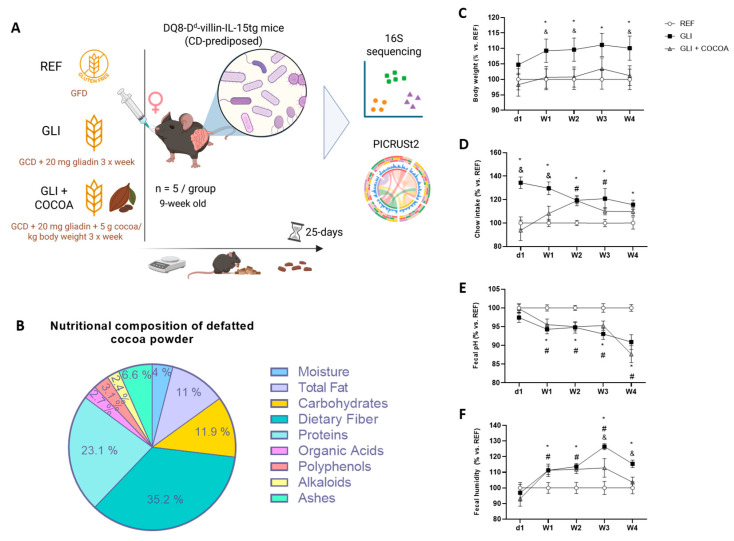
(**A**) Experimental design used to evaluate in vivo the effect of cocoa administration alongside a GCD in the gut microbial profile in a mouse model of CeD (n = 5/group). Animals either followed a gluten-free diet (REF), a gluten-containing diet supplemented thrice a week by a 20 mg gliadin ball (GLI), or the latter with an additional supplementation of defatted cocoa at 5 g/kg body weight 10 min prior to gliadin administration (GLI + COCOA). (**B**) Nutritional composition of the defatted cocoa powder was administered to the GLI + COCOA groups as an adjuvant to a GCD. Values are expressed per 100 g of total cocoa weight. Body weight (**C**), chow (**D**), fecal pH (**E**), and humidity (**F**) throughout the 25-day nutritional intervention. * *p* ≤ 0.05 GLI vs. REF, # *p* ≤ 0.05 GLI + COCOA vs. REF, and & *p* ≤ 0.05 GLI + COCOA vs. GLI.

A total of 15 nine-week-old female mice (5 per group) were randomly assigned to three experimental groups (Figure 1A). Sample size was calculated using the Granmo software v8.0 (https://www.datarus.eu/aplicaciones/granmo/, accessed on 10 April 2023), using intestinal anti-gliadin IgA as the primary endpoint (based on prior results showing that 10% of healthy animals and 90% of celiac animals displayed this variable), assuming no dropouts, a two-sided type I error of 0.05 and an allocation ratio of 1. The healthy control group (REF) received the GFD and 100 μL of phosphate-buffered saline (PBS, pH 7.2) via oral gavage, using a Hamilton syringe and a 22 G gavage tube, to replicate handling conditions. The disease control group (GLI) was fed a standard gluten-containing diet (GCD; Teklad 2018 Global 18% Protein Rodent Diet, Inotiv, Lafayette, IN, USA) and additionally received thrice-weekly a 20 mg gliadin ball (Merck, Darmstadt, Germany) by oral gavage. The treatment group (GLI + COCOA) followed the same diet as the disease animals with an additional administration of defatted cocoa at 5 g/kg body weight (Idilia Foods, Barcelona, Spain) in PBS 10 min prior to the gliadin ball. No adverse effect or rejection behavior was observed after cocoa administration to mice, since the administered cocoa contained a 2.2% theobromine (a dose of 110 mg/kg) and a 0.2% caffeine (a dose of 10 mg/kg) and the acute oral lethal doses (LD_50_) were established as 1356 mg/kg and 180 mg/kg, respectively [23]. The nutritional composition of the administered cocoa can be found in Figure 1B. The human equivalent dose (HED) was calculated using body surface area normalization, yielding 0.4 g/kg for a 60 kg adult [24]. This would translate into a dose of 24 g of defatted cocoa thrice a week, which aligns with previous clinical interventions with doses ranging from 10 to 26 g/daily [25,26]. Throughout the 25-day-long intervention, multiple physiological and morphological variables were tracked, including animal weight, chow, water intake, fecal pH, and humidity.

### 2.2. Sampling

On the 25th day of intervention (d25) animals were anesthetized intramuscularly with 90 mg/kg of ketamine (Merial Laboratories S.A. Barcelona, Spain) and 10 mg/kg of xylazine (Bayer A.G., Leverkusen, Germany) and exsanguinated. Multiple biological samples, including blood and distal small intestinal (dSI) fragments were collected and stored for future analysis. The content of the cecum was obtained and stored to characterize and predict the biological functions of its microbial community.

### 2.3. Taxonomic Profiling of the Cecal Microbiota

Characterization of the cecal microbiota was performed using female animals (*n* = 5) in Microomics Systems S.L (Barcelona, Spain). Microbial community composition was analyzed by amplifying and sequencing the V3–V4 regions of the 16S rRNA gene using Illumina MiSeq (2 × 300 bp) (Illumina, San Diego, CA, USA). Raw demultiplexed reads were processed with QIIME2 v. 2024.10.1 [27] and DADA2 v. 1.36.0 [28] to generate phylotype/operational taxonomic units (OTUs) data. Data on phylotype and OTUs were used to compute α-diversity (richness and evenness) and β-diversity (Bray–Curtis distance). Richness and evenness were analyzed with a generalized linear model (GLM) or a mixed GLM (R Packages MASS v. 7.3.65 [29] and NBZIMM v. 1.0 [30], for richness, and glmmTMB v. 1.1.10 [31] and betareg v. 3.2-1 [32] for evenness). Principal component analysis (PCA) and ordination plots were generated in R (v4.2.0), and group differences were tested with PERMANOVA, ANOSIM, and PERMDISP [33]. Phylotypes were taxonomically assigned using a Bayesian Classifier [34] trained on the SILVA v138 database [35]. Taxa under 1% relative abundance were grouped as “<1%” in stacked bar plots. Differential abundance was tested via Negative Binomial GLMs (MASS or NBZIMM). Only taxa species showing at least one statistically significant pairwise difference were represented in the boxplots.

### 2.4. Venn Diagrams

Venn diagrams were constructed to visualize the number of shared and unique taxa among the experimental groups at both the genus and species levels. A taxon was considered to be present in a group if it was detected in at least 80% of samples within that specific condition. Presence–absence matrices were generated based on the occurrence of each taxon within groups, and intersections were computed using R program v. 4.5.1 (R Core Team (2023) R: A Language and Environment for Statistical Computing_, R Foundation for Statistical Computing, Vienna, Austria) and Rstudio v. 2025.09.1 (RStudio Team (2023), RStudio: Integrated Development for R. RStudio, PBC, Boston, MA, USA) and the VennDiagram v. 1.8.2 and ggvenn v. 0.1.19 packages. The resulting plots illustrate overlapping and distinct microbial members between groups.

### 2.5. Linear Discriminant Analysis (LDA)

To identify bacterial taxa that best explained the differences among experimental groups, an LDA was performed on the relative abundance data at both the genus and species levels. The analysis was conducted in R/R Studio using the MASS v. 7.3.65 package. Prior to the analysis, unidentified and uncultured taxa were excluded to reduce noise and improve model robustness. LDA models were built separately for each pairwise comparison between experimental groups. The resulting discriminant functions were used to assess group separation and to determine which taxa contributed most strongly to the observed differences. LDA coefficients were visualized as horizontal bar plots generated with ggplot2 v. 4.0.0, where positive and negative scores indicated taxa associated with each respective group.

### 2.6. Autoantibody-Microbiota Correlations

CeD is characterized by the induction of several types of autoantibodies (autoAbs), including anti-gliadin and anti-transglutaminase 2 (TG2). Hence, levels of anti-gliadin and anti-TG2 autoAbs from the IgA and IgG isotypes were quantified in plasma and intestinal samples [36]. AutoAb levels were correlated with microbial abundance data, considering both all observed OTUs and all detected bacterial genera, using R/R Studio and the broom, Hmisch, and pheatmap packages. Correlations were computed across all animals, as well as within each treatment group separately. Within the heatmap, the red colour indicates positive correlations and the blue colour indicates negative ones. Significant correlations are marked with asterisks in the heatmap. Linear regressions are only displayed for those negative correlations showing statistical significance for the GLI + COCOA group with the Spearman test.

### 2.7. Goblet Cell Quantification

Due to changes in mucin-related taxa, goblet cells were also studied. In order to quantify the amount of goblet cells per intestinal villi, periodic acid–Schiff (PAS) staining of intestinal samples was performed as previously described [37]. Briefly, dSI segments were fixed in PBS-paraformaldehyde 4% (Applichem Panreac ITW Reagents, Castellar del Vallès, Spain), dehydrated, embedded in paraffin, and sectioned at 5 µm. Sections were deparaffinized, rehydrated, and stained with periodic acid–Schiff (PAS) (Merck) and Mayer’s hematoxylin (Merck, Darmstadt, Germany). After counterstaining and mounting, slides were examined using the bright-field microscope Olympus BX41 (Olympus Corporation, Shinjuku, Tokyo, Japan). Goblet cells per villous were identified based on PAS-positive (magenta) mucin staining using the Image J software v. 1.54s6 (Image Processing and Analysis in Java, National Institute of Mental Health, Bethesda, MD, USA). The sample size was five animals per treatment and five villi per animal.

### 2.8. Functional Profiling of the Cecal Microbiota

Gut microbial functional profiles were predicted with Phylogenetic Investigation of Communities by Reconstruction of Unobserved States (PICRUSt2) [38] by placing phylotypes into a reference tree (20,000 16S rRNA genes from Integrated Microbial Genomes (IMG) database) and annotated using Kyoto Encyclopedia of Genes and Genomes (KEGG) orthologs (KO) [39]. A significance threshold of 0.05 was used throughout the analyses. In each pairwise comparison, results express the enrichment of the first group in comparison to the second one.

### 2.9. Statistical and Data Analysis

Statistical analyses were conducted using IBM SPSS Statistics for Windows, Version 29.0.2.0 (IBM Corp., Armonk, NY, USA). The Shapiro–Wilk test was applied to assess data normality, and the Levene test was used to evaluate homogeneity of variances. For datasets meeting both assumptions of normality and homogeneity, one-way or two-way ANOVA followed by Dunnett’s T3 post hoc test was performed. For datasets that did not meet the previous assumptions, the non-parametric Kruskal–Wallis test was applied, followed by pairwise post hoc comparisons using Dunn’s test with Bonferroni correction for adjusted significance. Results are presented as mean ± SEM or as mean percentages with respect to the REF animals (designed as 100%) ± SEM, unless stated otherwise.

## 3. Results

### 3.1. Follow-Up

The analysis of the body weight, chow intake, fecal humidity, and fecal pH evolution throughout the 25-day intervention showed significant differences between the REF and GLI groups (Figure 1C–F). While animal body weight was significantly higher in gliadin-consuming animals than in the healthy group, normalization of the expected weight gain observed in the REF group to a 100%—since mice were still in the growth phase—showed that cocoa administration was able to counteract the weight gain associated with gliadin intake throughout the duration of the whole study (Figure 1C). Conversely, chow intake was initially higher in GLI animals, but this behavior was normalized over the course of the initial two weeks of the experimental period (Figure 1D). Fecal pH was significantly lower in both gluten and gluten + cocoa-consuming animals compared to those in a GFD (Figure 1E), while the increase in fecal humidity observed in GLI animals was only partially prevented by cocoa administration in the last two weeks of the intervention (Figure 1F).

### 3.2. Microbial Diversity Profile

**Figure 2:**
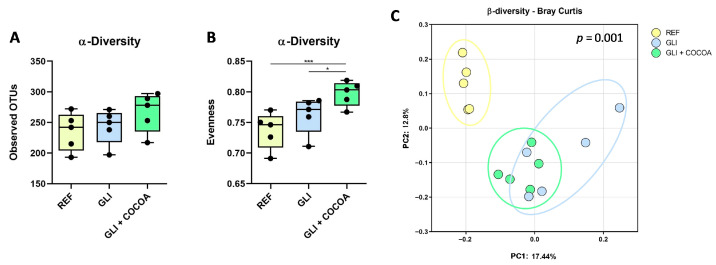
Alpha-diversity of the cecal microbiota expressed as (**A**) observed operational taxonomic units (OTUs) and (**B**) and Pielou’s evenness. (**C**) Principal component analysis (PCA) of the β-diversity of the cecal microbiota using the Bray–Curtis distance (n = 5/group). Colored ellipses indicate 95% confidence intervals around the β-diversity centroids of each group, showing the clustering of REF (yellow), GLI (blue), and GLI + COCOA (green) samples. * *p* ≤ 0.05 and *** *p* ≤ 0.001.

While no difference was found between groups for the α-diversity in terms of observed OTUs (Figure 2A), the group with cocoa intake displayed higher Pielou’s evenness index than the remaining groups (Figure 2B), indicating a higher similarity in proportions among the species present in that group. As for the β-diversity (Figure 2C), significant differential clustering was found between REF-GLI (*p* = 0.0135), REF-(GLI + COCOA) (*p* = 0.0135), and GLI-(GLI + COCOA) (*p* = 0.034).

**Figure 3:**
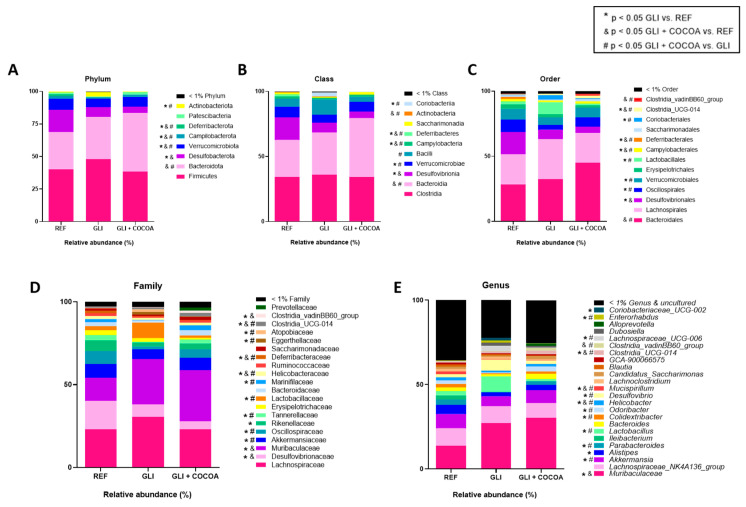
Taxonomic composition of the cecal microbiota (n = 5/group). Stacked bar plots show the relative abundance (%) of bacterial taxa at six taxonomic levels: phylum (**A**), class (**B**), order (**C**), family (**D**), and genus (**E**), across experimental groups (REF, GLI, and GLI + COCOA). Taxa with a relative abundance <1% or unidentified are grouped together. Data represent the mean relative abundance per group. Symbols indicate statistically significant differences: * *p* ≤ 0.05 GLI vs. REF, & *p* ≤ 0.05 GLI + COCOA vs. REF, and # *p* ≤ 0.05 GLI + COCOA vs. GLI.

**Figure 4:**
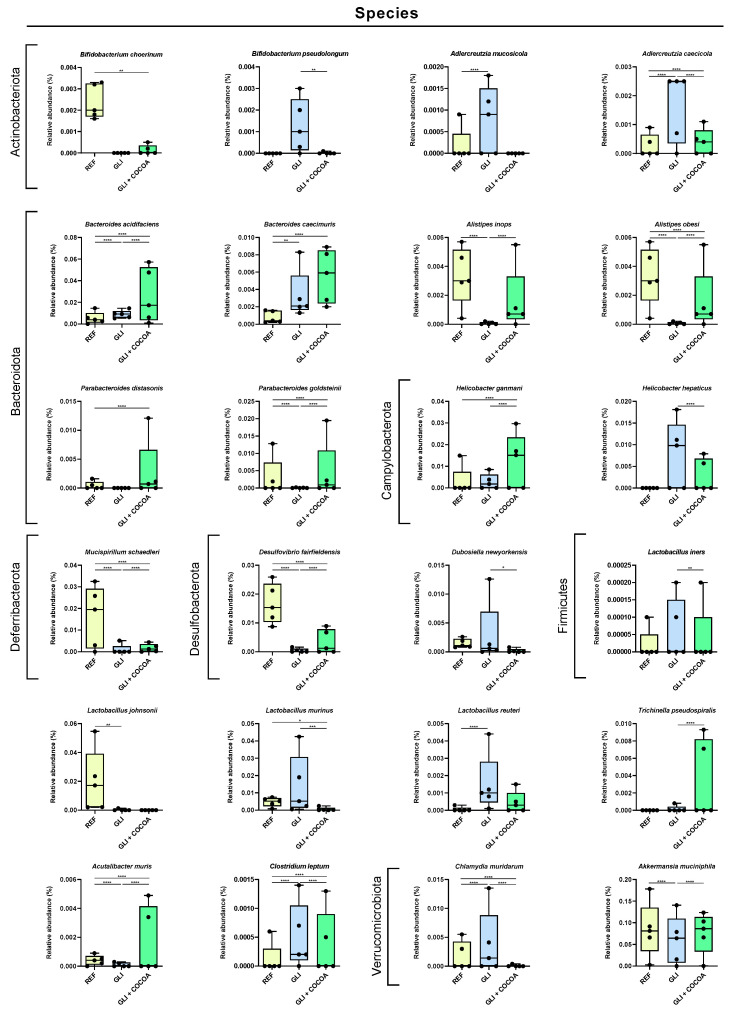
Relative abundance (%) of the species present in the cecal microbiota of DQ8-D^d^-villin-IL-15tg mice (n = 5/group) at d25 of the intervention. * *p* ≤ 0.05, ** *p* ≤ 0.01, *** *p* ≤ 0.001, and **** *p* ≤ 0.0001.

In terms of phyla (Figure 3A and Figure 4), addition of cocoa to a GCD prevented the increase in the relative abundance of Actinobacteriota and the decrease in Verrucomicrobiota associated with gliadin intake. Among microbial genera (Figure 3E), cocoa prevented the increase in *Lactobacillus*, *Desulfovibrio*, *Lachnospiraceae_UCG-006*, *Enterorhabdus*, and *Coriobacteriaceae_UCG-002* associated with gliadin intake, and likewise, the decrease in *Akkermansia*, *Parabacteroides*, *Colidextribacter*, *Odoribacter*, *Helicobacter*, and *Candidatus Saccharimonas*.

At the species level, a GCD led to a decrease in the relative abundance of *Bifidobacterium choerinum*, *Alistipes inops*, *A*. *obesi*, *Parabacteroides goldsteinii*, *Mucispirillum schaedleri*, *Desulfovibrio fairfieldensis*, and *Akkermansia muciniphila*, which were inhibited by cocoa intake (Figure 4). Moreover, the abundance increase in the GLI group of *B*. *pseudolongum*, *Adlercreutzia mucosicola*, *A*. *caecicola*, *Clostridium leptum,* and *Chlamydia muridarum* was effectively prevented by addition of cocoa into a GCD in the context of CeD. Alternative species whose relative abundance was modified by cocoa intake were the beneficial bacteria *Lactobacillus gasseri*, *Bacteroides acidifaciens*, and *B*. *caecimuris*, which were increased, and the opportunistic or pathogenic bacteria *Helicobacter hepaticus* was decreased. A heatmap clustering of genera (Supplementary Figure S1) showed a clear unsupervised grouping of samples based on treatment group. Moreover, for most of the samples, higher similarity was found between the REF and GLI + COCOA groups, than between the GLI and GLI + COCOA groups, as observed by sample distribution within the heatmap.

### 3.3. Microbial Presence Analysis

**Figure 5:**
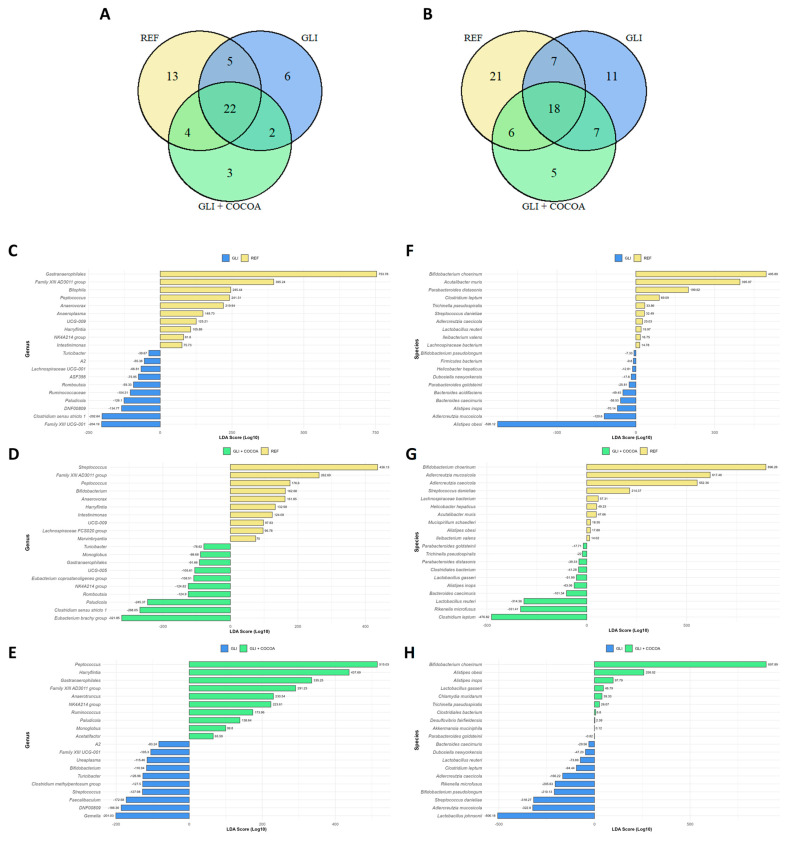
(**A**,**B**) Venn diagram showing shared and unique gut microbial taxa among treatment groups (n = 5/group). The diagram illustrates the presence of bacterial genera (**A**) or species (**B**) across the three experimental groups: REF, GLI, and GLI + COCOA. Each circle represents one treatment group, and overlapping areas indicate taxa detected in multiple groups, while non-overlapping areas represent unique taxa present only in that specific group. Only taxa detected in at least 80% of samples per group were included. (**C**–**H**) Linear discriminant analysis (LDA) plot comparing the relative abundance of genera (**C**–**E**) or species (**F**–**H**) between the following groups: REF-GLI (**C**,**F**), REF-GLI + COCOA (**D**,**G**), and GLI-GLI + COCOA (**E**,**H**). Taxa overrepresented in the REF group are depicted yellow, in the GLI group are blue, and in the GLI + COCOA group are green.

At the genus level, gut microbial presence or absence of specific bacterial groups was influenced by dietary interventions (Figure 5A and Supplementary Table S1). This analysis was made through a Venn diagram, which codes for the presence or absence of specific taxa. Across all groups, 22 genera were shared, representing a core microbiota maintained regardless of diet. However, distinct shifts were observed in response to gluten exposure and cocoa supplementation. In mice fed a gluten-containing diet (GLI), six genera were unique (*Coriobacteriaceae UCG-002*, *Clostridia UCG-014*, *Roseburia*, *Eubacterium xylanophilum* group, *Eubacterium coprostanoligenes* group, and an uncultured genus from the Eggerthellaceae family), reflecting a microbial dysbiosis associated with gliadin intake which cocoa was able to counteract. Two more genera were associated with gliadin intake (i.e., *UCG-010* and *Family XIII UCG-001*), the appearance of which was not prevented by cocoa administration. Simultaneously, the addition of cocoa to the GCD (GLI + COCOA) resulted in three unique genera (*Bilophila*, *Candidatus Saccharimonas*, and an unspecified genus from the Ruminococcaceae family), which could be explained by the prebiotic effect exerted by some components present in cocoa (i.e., fiber and procyanidins). Furthermore, four genera were shared exclusively between the REF and GLI + COCOA groups (*Erysipelatoclostridium*, *Ureaplasma*, *Oscillibacter* and *Akkermansia)*, but absent in GLI, indicating that cocoa supplementation partially preserved the microbiota profile characteristic of the healthy animals.

At the species level, dietary treatments also differently shaped the gut microbial composition (Figure 5B and Supplementary Table S2). A set of 18 species were consistently detected in all diets, forming a core microbiota that remained stable regardless of intervention. Beyond this shared core, notable differences emerged. Mice receiving a GCD harbored 18 differentiated species (e.g., *Lactobacillus reuteri* or uncultured *Coriobacteriales*) out of which only 7 were still present after addition of cocoa into the diet (e.g., *Bacteroides acidifaciens*). Cocoa intake also promoted the growth of five different species which appeared exclusively in this group (e.g., various uncultured Clostridia). Interestingly, six species were found only in REF and GLI + COCOA, but not in GLI, indicating that cocoa helped preserve features typical of the gluten-free condition (e.g., *Akkermansia muciniphila*). Conversely, seven species were common to GLI and GLI + COCOA but absent in REF, while six species were shared between REF and GLI but missing in GLI + COCOA, reflecting intermediate overlaps among the three dietary patterns. A list of all shared and exclusive taxa within each group and comparison can be found in Supplementary Tables S1 and S2. These findings suggest that cocoa exerts a preventive effect against gliadin-induced microbial dysbiosis, helping maintain a gut microbial composition closer to that of healthy, gluten-free controls.

### 3.4. Discriminant Taxa Identification

LDA revealed marked shifts in gut microbiota composition associated with gliadin intake and the modulatory effect of cocoa supplementation. For each pairwise comparison, the ten taxa displaying the highest (and lowest) LDA score were included. Interestingly, four genera were overrepresented in both the REF and GLI + COCOA groups in their pairwise comparison with GLI animals, namely *Gastranaerophilales*, *Family XIII AD3011 group*, *Peptococcus*, and *NK4A214* (Figure 5C,E). These anaerobic, Gram-positive bacteria primarily ferment amino acids and peptides rather than carbohydrates, producing short-chain fatty acids (SCFAs) such as acetate, propionate, and sometimes branched-chain fatty acids [40]. In terms of species, only two of them overlapped as driving factors for both REF and GLI + COCOA groups (Figure 5F,H), indicating that cocoa supplementation partially mitigated gliadin-induced dysbiosis, reducing the dominance of Bacteroidota (*Parabacteroides goldsteinii*, *Bacteroides acidifaciens*, *B*. *caecimuris*, *Alistipes inops*, and *A*. *obesi*) while promoting taxa linked to gut health, notably *Akkermansia muciniphila* and *Lactobacillus reuteri* (Figure 5G,H). Although the GLI + COCOA profile did not fully revert to REF composition, the introduction of mucin-degrading and fiber-associated bacteria suggests a protective role of cocoa polyphenols in restoring microbial balance and enhancing intestinal barrier function.

### 3.5. Autoantibody–Microbiota Correlations

**Figure 6:**
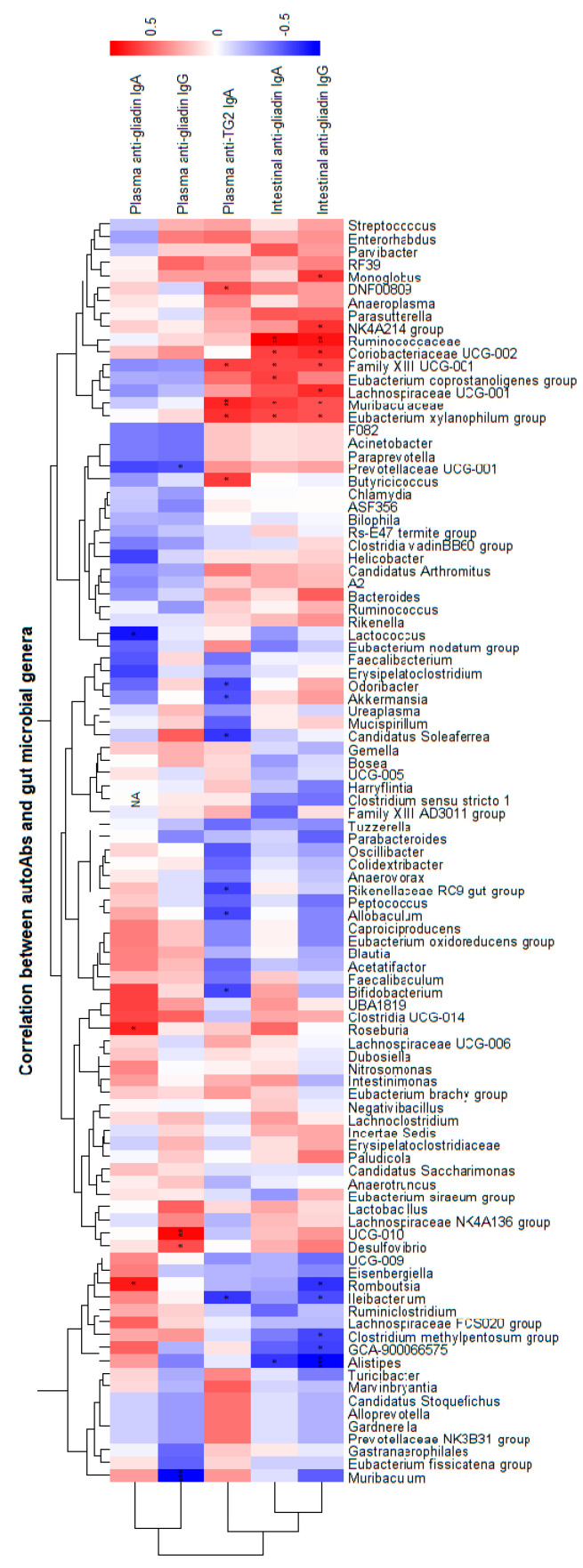
Heatmap showing correlations between gut microbial genera and autoantibody levels (anti-gliadin and anti-transglutaminase 2 (TG2)) of the IgA and IgG isotypes measured in plasma and intestinal samples (n = 5/group). Spearman correlation coefficients are represented by color, with blue indicating negative correlations and red indicating positive correlations. Only correlations that were statistically significant (*p* < 0.05) are marked with asterisks (*). White or neutral cells indicate non-significant correlations or taxa/autoantibodies with insufficient variation. * *p* ≤ 0.05, ** *p* ≤ 0.01, and *** *p* ≤ 0.001.

Multiple significant positive and negative correlations were found between autoAb levels (disease biomarkers) and relative abundance of microbial taxa, both when assessing animals regardless of group and separately (Figure 6 and Supplementary Figure S2). In gluten-consuming animals, the genera with stronger positive associations to autoAb levels were *UCG-010*, *RF39*, *Muribaculaceae*, *Harryflintia*, and *Akkermansia* (Supplementary Figure S2A). Cocoa administration led to significant negative correlations between the relative abundances of various taxa and the autoAb levels (Supplementary Figure S2B).

**Figure 7:**
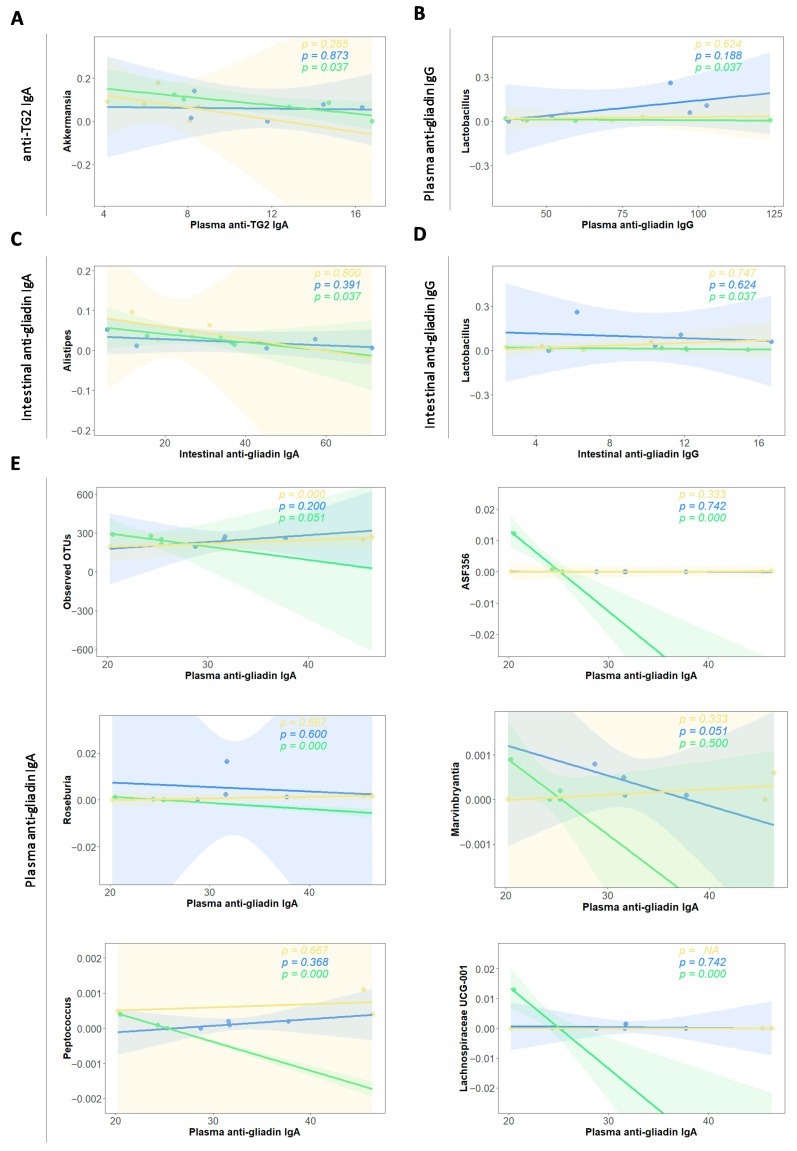
Linear regression plots illustrating the association between specific gut microbial genera and autoantibody levels in the context of cocoa intake (n = 5/group). Each panel shows the relative abundance of genera that were negatively correlated with anti-transglutaminase (TG2) IgA (**A**), plasma anti-gliadin IgA (**B**) or IgG (**C**), or intestinal anti-gliadin IgA (**D**) or IgG (**E**), specifically in the GLI + COCOA group. Spearman correlation coefficients were used to identify these associations and regression lines and confidence intervals are used to indicate the strength and direction of the correlation, highlighting the potential protective effect of cocoa on CeD-associated autoimmunity. “NA” *p*-values indicate insufficient variability within the group to compute the correlation. Only genera whose negative correlations were statistically significant in the group-specific analysis are shown.

Plasma levels of anti-TG2 IgA (Figure 7A) were significantly negatively correlated with the relative abundances of *Akkermansia* (*p* = 0.037) and plasma anti-gliadin IgA levels (Figure 7B) with the number of observed OTUs (*p* = 0.051) and with the relative abundance of the genera *ASF356* (*p* < 0.001), Roseburia (*p* < 0.0001), *Peptococcus* (*p* < 0.0001), *Lachnospiraceae UCG-001* (*p* < 0.0001), and *Marvinbryantia* (*p* = 0.041). Plasma and intestinal anti-gliadin IgG (Figure 7C,E) levels from the GLI + COCOA group were negatively correlated with the relative abundance of the genera *Lactobacillus* (*p* = 0.037), while intestinal anti-gliadin IgA (Figure 7D) levels displayed a negative correlation to the relative abundance of the genera *Alistipes* (*p* = 0.037).

### 3.6. Quantification of Goblet Cells

**Figure 8:**
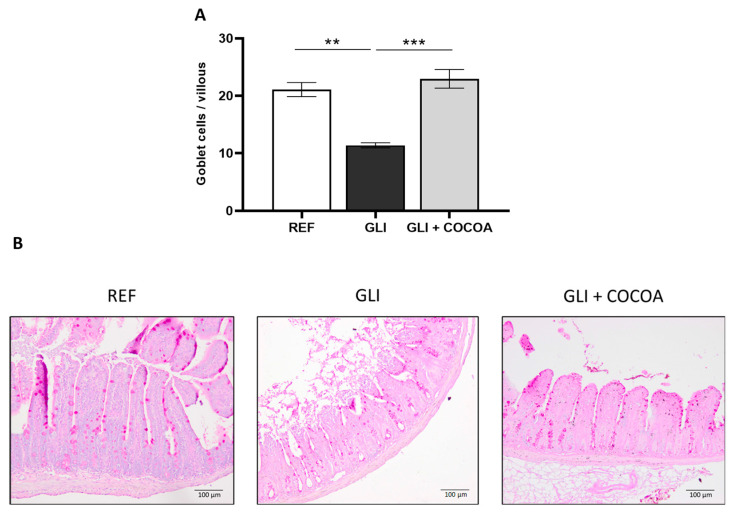
Quantification of goblet cells per villus in distal small intestinal villi using periodic acid-Schiff (PAS) immunohistochemistry staining (n = 5/group) (**A**). Data represent mean values ± SEM obtained from five animals, with five villi analyzed per animal. ** *p* ≤ 0.01 and *** *p* ≤ 0.001. (**B**) Representative microscopic images of the PAS-stained dSI sections used to visualize goblet cells. Magnification 100×.

After observing changes in the abundance of the mucin-degrading bacteria *Akkermansia*, the mucin-producing goblet cells were studied to evaluate if their presence in intestinal villi differed based on treatment group. PAS staining of intestinal samples revealed marked differences in goblet cell numbers among the experimental groups (Figure 8A,B). Gliadin consumption resulted in a significant reduction in goblet cells per villus compared with the reference group. In contrast, supplementation of cocoa alongside gliadin resulted in a preventive effect on the loss of goblet cells, maintaining levels similar to the REF group. This demonstrates a strong protective effect of cocoa on mucin-producing cell populations in the dSI.

### 3.7. Predicted Microbial Functions

**Figure 9:**
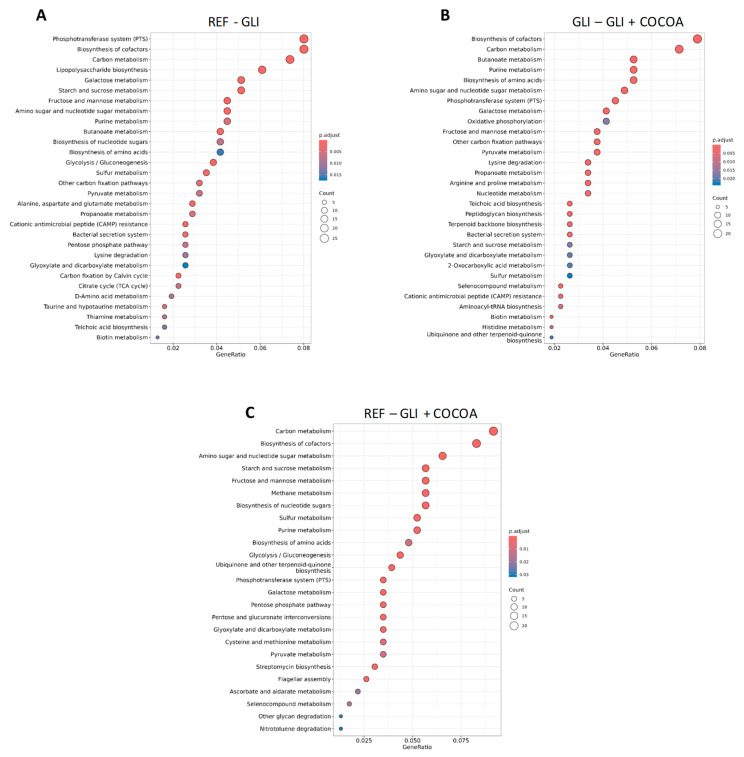
Overrepresented Kyoto Encyclopedia of Genes and Genomes (KEGG) functions, i.e., top 30 more significant, lowest *p*-value pathways, based on diet effect (*p* < 0.05) (n = 5/group): REF vs. GLI (**A**), GLI vs. GLI + COCOA (**B**), or REF vs. GLI + COCOA (**C**). Pathways are always enriched in the first group. Dot color reflects *p*-value, with red meaning higher and blue lower significance, and size indicates the number of functions in each pathway. GeneRatio is calculated based on the number of annotated functions in a pathway (count) as a fraction of total functions of interest.

Metataxonomic analyses elucidated potential functional pathways enriched or hindered in each group, comparatively. First, comparison of pathways enriched in REF vs. GLI showed that the gluten-free animals had a microbiota profile functionally enriched in carbohydrate metabolism (e.g., carbon, starch and sucrose, fructose and mannose, amino and nucleotide sugar metabolism, pentose phosphate pathways, and glycolysis/gluconeogenesis), while amino acid biosynthesis was more favored in the exclusively gluten-consuming (GLI) microbiota (pyruvate, alanine, aspartate, glutamate, lysine, D-amino acid, taurine, and hypotaurine metabolism) (Figure 9A).

While this observation is consistent with the fact that gluten is a protein, and as such, the GLI group consumes a diet enriched in amino acids, GLI animals also displayed a microbiota enriched in pathways related to secretion systems (bacterial secretion system), and cell wall synthesis (peptidoglycan and teichoic acid biosynthesis), pointing to a different microbial structure with increased fermentative capacity and perhaps inflammation-associated functions (e.g., sulfur metabolism), although direct inflammatory effects were not assessed in this study. Finally, cocoa supplementation (GLI + COCOA) was capable of limiting some of the functional shifts caused by gluten. Compared to GLI (Figure 9B), its microbial function profile was less enriched in biosynthetic and inflammatory pathways, but it did not fully restore the functional enrichment seen in REF mice. Identification of the pathways underrepresented in both REF-GLI + COCOA and GLI-GLI + COCOA plots indicated that cocoa supplementation led to enrichment of pathways related to amino acid and nitrogen metabolism (histidine, lysine, alanine, aspartate, glutamate, taurine, hypotaurine, D-amino acid, and tryptophan metabolism), reflecting the additional protein intake derived from the GCD, and anti-oxidant/redox metabolism (ascorbate, aldarate, biotin, glyoxylate and dicarboxylate metabolism, or ubiquinone and other terpenoid-quinone biosynthesis) (Figure 9C).

**Figure 10:**
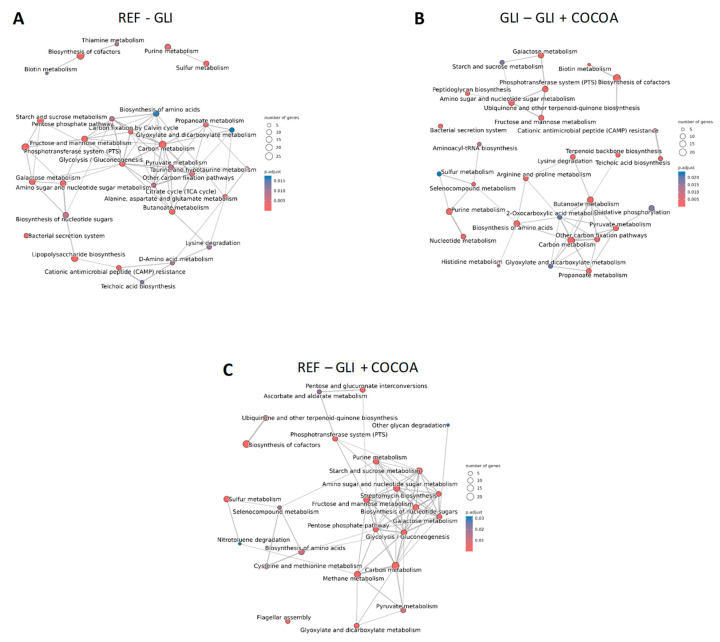
Enrichment map showing the most overrepresented pathways in the first of the two groups: REF vs. GLI (**A**), GLI vs. GLI + COCOA (**B**), and REF vs. GLI + COCOA (**C**). Edges join two pathways if there is a similarity of at least 5%. Edges are sized on the basis of the number of genes shared by the connected pathways. The circle size (gene count) reflects the number of gene orthologues belonging to a specific pathway which are overrepresented in the first group as compared to the second one. The color scale indicates significance, with the lowest significance being represented by blue tones (*p* ≤ 0.05) and the highest one by red (*p* ≤ 0.001). (n = 5/group).

A more detailed visualization of the enriched pathways and their shared genes or the specific gene orthologues participating in some of the most enriched pathways for each pairwise comparison is depicted through an enrichment map (Figure 10A–C) and category net plot graph (Supplementary Figure S3A–C), respectively. A heatmap showing clustering of enriched KEGG pathways (y axis) based on diet (x axis), again demonstrated a clear differentiation based on treatment group (Supplementary Figure S4).

## 4. Discussion

It has been confirmed for some time now that there is a deep relationship between CeD pathogenesis and the gut microbial ecosystem of patients suffering from this disease. Although causality still needs to be verified, i.e., is the microbial imbalance a cause or a consequence of the disease, there is no doubt that intestinal dysbiosis is a trademark of CeD. While no characteristic microbial signature has been defined yet for this disease, previous studies have frequently reported an increase in the abundance of the phyla Proteobacteria [41,42,43,44,45,46] and Bacteroidetes [44,45,47], while the abundance of Firmicutes [41,45,48,49,50] and Actinobacteriota [46,50] has been described to both increase and decrease as a result of CeD establishment (Table 1). While many changes have been reported at lower taxonomic levels, those which have been most frequently described include an increase in the abundance of opportunistic bacteria such as some strains belonging to the *Clostridium* [51] and *Helicobacter* [52] genera or a decrease in the health-associated genera *Akkermansia* [52,53], *Lactobacillus* [48,54,55], and *Bifidobacterium* [52]. On the line of pursuing the identification and characterization of a signature microbial profile characteristic of CeD and causative of the disease-associated intestinal dysbiosis and dysfunction, we report here for the first time the gut microbial composition of the novel DQ8-D^d^-villin-IL-15tg murine model of CeD—currently the model best suited to represent the complexity of CeD at the preclinical level—in the context of a GFD, a GCD, and a GCD supplemented with cocoa.

While it is not expected to find the exact same microbial profile in a murine model of a disease as in human patients, many of the previously reported changes in taxa in CeD have also been found in this study. Notably, a basal difference in the gut microbial profile is expected between the GFD and GCD groups, simply based on the fact that they consume a different diet. Nevertheless, the main driving factor for the observed changes is the inclusion of gluten into a diet administered to animals with a genetic predisposition to the development of the disease. Most importantly, addition of cocoa into a GCD administered to animals with a predisposition to CeD has been capable of preventing many of the gluten-derived changes in the abundance of microbial taxa. In the current model, CeD dysbiosis was characterized by an increase in the phyla Actinobacteriota and a decrease in Verrucomicrobiota, both of which were effectively prevented by cocoa intake. The same pattern was observed for multiple other genera and species, for which a gliadin-induced increase (e.g., *C*. *leptum*, *Lactobacillus*, *Roseburia*, *Ruminococcus*) or decrease (e.g., *Bifidobacterium*, *Bacteroides*, *Parabacteroides*, *Blautia*, *Akkermansia*) in abundance was prevented by cocoa. To this end, the crucial observation that cocoa is capable of partially reversing the break in microbial homeostasis and its subsequent dysfunction caused by gliadin intake in the context of CeD, and even more so, contribute to the increase in abundance of some recognized beneficial bacteria, is now very relevant. Globally, all of the results from this study contribute to reaffirm our hypothesis that the use of cocoa as an adjuvant to a GFD can be highly beneficial.

While previous studies have researched the gut microbial ecosystem in CeD and the effects exerted by polyphenols in this context [15], to our best knowledge no previous works have investigated the specific role of cocoa in CeD. Alternatively, only few prior studies have reported cocoa-derived effects on the gut microbial composition. On this line, two previous works on healthy [65] and orally sensitized rats [18] reported a decrease in the abundance of Firmicutes and an increase in the abundance of Bacteroidetes after cocoa intake, results which align with the findings of the current project. Simultaneously, while a decrease in the abundance of *Lactobacillus* [48,54,55] has been more frequently associated with CeD than the opposite effect, an increase in this genus, as found in the current project, has also been previously reported [45]. Interestingly, administration of cocoa prevented this increase in the abundance of *Lactobacillus*, a phenomenon which has been reported before for cocoa-derived polyphenols [66] and which could be explained by cocoa’s selective antimicrobial activity against Gram-positive bacteria [67]. Moreover, the phyla Firmicutes and Actinobacteriota, as well as some of its relevant genera such as *Lactobacillus*, *Clostridium*, or *Bifidobacterium*, which display this phenotype, have all been observed to decrease in the cocoa-consuming animals. Even so, an increase in some beneficial bacteria belonging to these genera, as observed in the current project for *L*. *gasseri*, *B*. *acidifaciens*, and *B*. *caecimuris*, has also been previously observed after cocoa intake for species such as *L*. *reuteri* or *B*. *uniformis* [18]. Most interestingly, it was previously reported that children at a high risk of developing CeD exhibited higher levels of specific Bacteroidetes species, including *B*. *vulgatus*, while those with a low risk displayed increased amounts of *B*. *uniformis*, among others [42].

In terms of CeD, a higher abundance of sulphate-reducing bacteria like *C*. *histolyticum* [56] has been observed. This aligns well with our results, where increased abundance of the genus *Desulfovibrio* was observed, a phenomenon that was effectively prevented by addition of cocoa into the gliadin-containing diet. Similarly, while a decrease in *Parabacteroides* has previously been reported in the context of CeD [62], this was prevented by cocoa intake alongside gluten. Another relevant observation was the preventive effect exerted by cocoa on the increased abundance of opportunistic bacteria such as *Chlamydia muridarum* or *Helicobacter hepaticus* linked to gliadin intake, a phenomenon which has previously been reported in CeD [68].

A decrease in *A*. *muciniphila* has been previously associated with the development of CeD [52,53]. Interestingly, in the current study, cocoa was linked to an increase in the relative abundance of these recognized beneficial bacteria, which degrade mucin and produce the SFCAs acetate and propionate, which can then be taken up by alternative species in order to generate butyrate. While previous studies have not reported changes in the abundance of *A*. *muciniphila* after cocoa intake, the prebiotic role of cocoa (mainly due to its high content in fiber and polyphenols) has been demonstrated in previous works [65]. Moreover, since this species primarily consumes intestinal mucin, a mutualistic relationship has been previously established between *A*. *muciniphila* and goblet cells, as its presence leads to an increased production of mucin by goblet cells in order to maintain a stable mucous layer. SCFAs also contribute to this relationship since they support goblet cell differentiation and promote gut barrier integrity [69]. Hence, our finding that in the cocoa-consuming animals not only an increased abundance of *A*. *muciniphila*, but also increased levels of goblet cells per villi exist can be indicative of a role exerted by cocoa in amelioration of the gliadin-induced intestinal dysregulation.

The detection of autoAbs against the TG2 enzyme, gliadin, or deamidated gliadin peptides are the first steps in the clinical diagnosis of CeD [70,71]. These autoAbs were previously quantified in plasma and intestinal samples [36]. Given the hypothesis that the gut microbiota may contribute to the loss of oral tolerance to gluten or to immune activation in the context of celiac disease [72], examining correlations between the abundance of specific microbial taxa and autoAb levels could provide insights into the role of the gut microbiota in CeD pathogenesis. Most interestingly, a negative correlation between the number of observed OTUs in the cocoa-consuming animals and the levels of anti-gliadin IgA antibodies in plasma samples, similar to previous results reporting a negative correlation between observed OTUs and anti-TG2 IgA antibodies in CeD [59] was found. Simultaneously, the abundance of several genera in the microbiota of cocoa-consuming animals, including *Akkermansia*, *Roseburia*, or *Lactobacillus*, was negatively correlated to the generation of autoAbs (anti-TG2 IgA, and anti-gliadin IgA and IgG, respectively) indicating a protective effect exerted by specific cocoa-derived taxa on one of the most clinically relevant biomarkers of CeD. This aligns with the results reported in a previous study using an intolerance model, where addition of cocoa into the diet resulted in a significant decrease in intestinal IgA antibodies [18]. While the pathway behind this effect is not elucidated, a modification of the gut microbiota as described by the authors could be explanatory of the tolerogenic effect exerted by cocoa. Nevertheless, given the study design, we cannot exclude that the observed effects reflect a combination of direct immunomodulatory actions of cocoa and indirect effects mediated by modulation of the gut microbial ecosystem.

In terms of inferred functionality of the gut microbiota, one of the main differences between the gluten-free and gluten-consuming groups was an increase in amino acid metabolism in the latter, an observation which has been reported before [73,74]. Moreover, CeD has also been associated with a decrease in vitamin and nutrient absorption [75], as observed in the current study by a significant enrichment in related metabolic pathways in animals following a GFD, compared to those in a GCD, including biosynthesis of cofactors, thiamine and biotin metabolism, pathways linked to vitamin metabolism, and carbon, galactose, starch and sucrose, fructose, and mannose metabolism, which are indicators of nutrient utilization. Alternative pathways that have also been linked to CeD are those associated with bacterial pathogenesis [76], and increased sulfur and nitrogen derivatives [76,77].

The pathways predicted to be enriched as a consequence of cocoa administration mainly demonstrate a modulation of the gut microbiota by cocoa’s polyphenols and bioactive compounds towards a microbial profile with increased capacity to metabolize phenolic compounds, vitamins, amino acids, and terpenoids. Interestingly, all of these pathways have been linked to beneficial health effects. A recent study researching the effect of cocoa supplementation with or without the addition of *Lactobacillus rhamnosus* in pigs reported an increase in the metabolism of terpenoids, amino acids, lipids, energy, vitamins, and cofactors in both groups on cocoa-containing diets [78]. Similarly, another work exploring the effect of cocoa administration in the context of colitis and alongside two different dietary interventions, also observed an increase in both carbohydrate, amino acid, and vitamin B6 metabolism [19]. Nevertheless, the same study reported that a typical Western diet led to a decrease in both nucleotide and terpenoid metabolic pathways. Since decreased absorption of some nutrients and vitamins has been reported for CeD, cocoa’s predicted ability to potentiate such pathways indicates a potential positive role in ameliorating CeD pathogenesis. Studies with other polyphenol-containing compounds, including blueberries in healthy [79] and obese [80] mice, tea in a model of antibiotic-induced dysbiosis [81], or walnuts in a mouse model of colitis [82], all led to a predicted increase in amino acid metabolism, as observed after cocoa administration in the current project.

Although this study included only females and a relatively small sample size, these choices were intentional: females were selected due to the well-established higher prevalence of CeD in females compared to males (2:1), and the sample size reflects the pilot nature of this project, aimed at characterizing for the first time the gut microbiota of this model and exploring the potential modulatory effects of cocoa. Estrous cycles were not synchronized and hormonal fluctuations were not controlled, which may have contributed to inter-individual variability and represents a limitation. Nevertheless, all mice were age-matched and maintained under identical housing and feeding conditions, which likely minimized some sources of variability; future studies should include males or both sexes to fully characterize the gut microbiota in this model. In addition, functional pathway analyses were based on PICRUSt2 predictions derived from 16S rRNA gene data and therefore reflect inferred microbial metabolic potential rather than direct measurements of gene expression, enzyme activity or metabolite concentrations, which were not assessed in this study. Importantly, the successful results obtained provide a strong foundation and emphasize the value of conducting further studies to validate and expand upon these findings.

First of all, the results of this study validate DQ8-D^d^-villin-IL-15tg mice as a suitable model to study CeD dysbiosis and impactful interventions to prevent it, specifically in female animals. Globally, the analyses performed in this study confirm that both microbial dysbiosis and dysfunction exist in females suffering from CeD. Moreover, the current work can help expand the knowledge on potential causative changes in taxa responsible for disruption of intestinal homeostasis and demonstrate that cocoa administration is capable of either preventing or mitigating them. Moreover, the current results show that cocoa also promotes the growth of beneficial bacteria beyond those taxa present in a GFD, due to its high content of polyphenols, as well as other bioactives like theobromine and fiber. Additionally, functional inference indicated that cocoa is capable of modulating the gut microbial function, enhancing pathways related to nutrient and vitamin metabolism. To this end, the current results demonstrate that the use of cocoa as an adjuvant to a GFD in the context of CeD could be promising to ameliorate CeD-associated dysbiosis, although further studies are required to validate these findings, as well as to ensure the same patterns are observed regardless of sex, and clarify its translational relevance in patients. In addition, the precise profile of cocoa polyphenols would help to ascertain the main contributor to this action, as well as the role of the other mentioned components such as theobromine or fiber.

## Figures and Tables

**Table 1:** Changes in microbial composition associated with CeD and comparison with results of the current study.

CeD (Literature)	GLI (vs. REF)	GLI + COCOA (vs. GLI)
↑/↓ Actinobacteria (Actinobacteriota) [50]/[46]	↑↑	↓↓
↓ *Bifidobacterium* [56]	↓	↑↑
↑ *B*. *angulatum* [57]	↑ *B*. *pseudolongum*	↓↓
↓ *B*. *longum* [58]	↓ *B*. *choerinum*	↑
↑/↓ Proteobacteria [41,42,43,44,45,46]/[50]	=	=
↑ *Helicobacter* [52]	↓↓	↑↑
↑ *Neisseria* [50]	NA	NA
↑ Bacteroidetes (Bacteroidota) [44,45,47]	↑	↑↑
↑/↓ *Alistipes* [59]/[60]	↓↓	↑
↑/↓ *Bacteroides* [51,61]	↓	↑
↑/↓ *B*. *vulgatus* [42]/[62]	↑↑ *B*. *acidifaciens*	↑↑
↑ *B*. *ovatus*, *B*. *plebeius*, *B*. *uniformis* [42]	↑↑ *B*. *caecimuris*	↑
↓ *Parabacteroides* [59]	↓↓	↑↑
↑ *Prevotella* [59]	NA	NA
↑/↓ Firmicutes [45,48,49]/[41,50]	↑	↓
↓ *Anaerostipes* [45]	NA	NA
↓ *Blautia* [45]	↓	↓
↑ *Clostridium* [51]	NA	NA
↓ *Clostridium histolyticum* [56]	↑↑ *C*. *leptum*	↓↓
↓ *Dialistes* [59]	NA	NA
↓ *Dorea* [52]	NA	NA
↓ *Faecalibacterium* [45]	=	=
↓ *Faecalibacterium prausnitzii* [56]	NA	NA
↑/↓ *Lactobacillus* [45]/[48,54,55]	↑↑	↓↓
↑ *Megasphaera* [52]	NA	NA
↑ *Roseburia* [51]	↑↑	↓↓
↓ *Roseburia intestinalis* [57]	NA	NA
↑/↓ *Ruminococcus* [59]/[45]	↑	↓↓
↑ *Staphylococcus* [43]	NA	NA
↑ *Staphylococcus hemolyticus* [42]	NA	NA
↓ *Staphylococcus aureus* [42,55]	NA	NA
Verrucomicrobiota	↓↓	↑↑
↓ *Akkermansia* [52,53]	↓↓	↑↑

Summary of studies reporting changes in microbial abundance in duodenal and fecal samples from active CeD patients compared to healthy controls, patients with non-active CeD or in a GFD [63,64]. For each taxon, a comparison was displayed between bibliographical findings and the results obtained in the current study. Arrows display non-significant (one arrow) or significant (two arrows) changes, an equal sign “=” indicates no variation between groups. NA = not applicable, i.e., the taxon was not detected in the current project.

## Data Availability

The original contributions presented in the study are included in the article and Supplementary Materials; further inquiries can be directed to the corresponding author.

## Supplementary Materials

The following supporting information can be downloaded at https://www.mdpi.com/article/10.3390/foods15020370/s1. Figure S1: Heatmap of scaled genus-level abundance in the cecal microbiota of mice under different dietary treatments. Each column represents an individual sample, and each row represents a bacterial genus grouped by phylum. Colors indicate the Z-score of relative abundance, with red showing higher and blue showing lower abundance relative to the mean. Samples are clustered based on similarity in microbial profiles, and are color-coded by diet: REF (red), GLI (blue), and GLI + COCOA (green). Dendrograms indicate hierarchical clustering of microbial communities based on Bray–Curtis dissimilarity; Figure S2: Heatmaps showing correlations between gut microbial genera and autoantibody levels (anti-gliadin and anti-transglutaminase 2 (TG2)) of the IgA and IgG isotypes measured in plasma and intestinal samples. Spearman correlation coefficients are represented by color, with blue indicating negative correlations and red indicating positive correlations. Only correlations that were statistically significant (*p* < 0.05) are marked with asterisks (*), * *p* ≤ 0.05, ** *p* ≤ 0.01, *** *p* ≤ 0.001. White or neutral cells indicate non-significant correlations or taxa/autoantibodies with insufficient variation. Heatmaps are shown separately for each experimental group: GLI (A) and GLI + COCOA (B); Figure S3: Category netplot depicting the linkages of the 5 most significant KEGG functions as a network to visualize which KEGG functions are involved in each of the enriched pathways and which of them are shared in pairwise comparisons between REF and GLI (A), GLI and GLI + COCOA (B) and REF and GLI + COCOA (C); Figure S4: Heatmap summarizing the functional analysis of the Kyoto Encyclopedia of Genes and Genomes (KEGG) pathways with statistically significant enrichment (*p* < 0.05). Rows represent individual KEGG pathways, and columns denote dietary treatment groups (REF, GLI, and GLI + COCOA). Color intensity reflects pathway expression/activity levels, with red indicating higher levels and blue representing lower levels. Hierarchical clustering of pathways and treatment groups is visualized through dendrograms, highlighting distinct functional patterns influenced by dietary interventions; Table S1: List of gut bacterial genera present in each section of the Venn diagram. The table summarizes the gut microbial genera detected in each experimental group (REF, GLI, and GLI + COCOA) and their intersections as shown in the corresponding Venn diagram. Genera listed in overlapping sections were detected in multiple groups, while those in non-overlapping sections are unique to that specific group. Only genera present in at least 80% of samples per group are included; Table S2: List of gut bacterial species present in each section of the Venn diagram. The table summarizes the gut microbial genera detected in each experimental group (REF, GLI, and GLI + COCOA) and their intersections as shown in the corresponding Venn diagram. Genera listed in overlapping sections were detected in multiple groups, while those in non-overlapping sections are unique to that specific group. Only genera present in at least 80% of samples per group are included.

## Abbreviations

The following abbreviations are used in this manuscript:

**Abbreviation**	**Meaning**
Ab	Antibody
CEEA	Ethics Committee on Animal Experimentation
CeD	Celiac disease
GCD	Gluten-containing diet
GFD	Gluten-free diet
GIP	Gluten immunogenic peptide
GLM	Generalized linear model
HED	Human equivalent dose
Ig	Immunoglobulin
KEGG	Kyoto Encyclopedia of Genes and Genomes
KO	KEGG ortholog
LDA	Linear discriminant analysis
MLN	Mesenteric lymph node
OTU	Operational taxonomic unit
PICRUSt2	Phylogenetic Investigation of Communities by Reconstruction of Unobserved States 2
SCFA	Short-chain fatty acid
SEM	Standard error of the mean
TG2	Transglutaminase 2
